# Weight and protozoa number but not bacteria diversity are associated with successful pair formation of dealates in the Formosan subterranean termite, *Coptotermes formosanus*

**DOI:** 10.1371/journal.pone.0293813

**Published:** 2023-11-13

**Authors:** Junyan Chen, Garima Setia, Li-Hsiang Lin, Qian Sun, Claudia Husseneder

**Affiliations:** 1 Department of Entomology, Louisiana State University Agricultural Center, Baton Rouge, Los Angeles, United States of America; 2 Department of Experimental Statistics, Louisiana State University Agricultural Center, Baton Rouge, Los Angeles, United States of America; Bayero University Kano, NIGERIA

## Abstract

New colonies of Formosan subterranean termites are founded by monogamous pairs. During swarming season, alates (winged reproductives) leave their parental colony. After swarming, they drop to the ground, shed their wings, and male and female dealates find suitable nesting sites where they mate and become kings and queens of new colonies. The first generation of offspring is entirely dependent on the nutritional resources of the founder pair consisting of the fat and protein reserves of the dealates and their microbiota, which include the cellulose-digesting protozoa and diverse bacteria. Since termite kings and queens can live for decades, mate for life and colony success is linked to those initial resources, we hypothesized that gut microbiota of founders affect pair formation. To test this hypothesis, we collected pairs found in nest chambers and single male and female dealates from four swarm populations. The association of three factors (pairing status, sex of the dealates and population) with dealate weights, total protozoa, and protozoa *Pseudotrichonympha grassii* numbers in dealate hindguts was determined. In addition, Illumina 16S rRNA gene sequencing and the QIIME2 pipeline were used to determine the impact of those three factors on gut bacteria diversity of dealates. Here we report that pairing status was significantly affected by weight and total protozoa numbers, but not by *P*. *grassii* numbers and bacteria diversity. Weight and total protozoa numbers were higher in paired compared to single dealates. Males contained significantly higher *P*. *grassii* numbers and bacteria richness and marginally higher phylogenetic diversity despite having lower weights than females. In conclusion, this study showed that dealates with high body weight and protozoa numbers are more likely to pair and become colony founders, probably because of competitive advantage. The combined nutritional resources provided by body weight and protozoa symbionts of the parents are important for successful colony foundation and development.

## Introduction

The Formosan subterranean termite, *Coptotermes formosanus* Shiraki (Blattodea: Rhinotermitidae), is a species in the family Rhinotermitidae, which belongs to the so-called lower termites (i.e., all termites except members of the family Termitidae). It is one of the most destructive and invasive termite species in the world, causing billions of dollars of damage to structures and crops globally each year [1, 2]. Like other subterranean termites, *C*. *formosanus* colonies consume only lignocellulose material, mainly in the form of wood [3]. Cellulose digestion by the termites’ endogenous enzymes alone is inefficient; therefore, subterranean termites have to collaborate with a complex community of gut symbionts consisting of protozoa, bacteria and archaea to ensure adequate nutrition [4, 5]. All gut protozoa and many prokaryote species are obligate, i.e., essential for survival in lower termites [4]. Protozoa provide cellulases [6–9] and proteases [10] to complement those of the termite and convert lignocellulose into acetic acid and short-chain fatty acids as energy source for the termites [4]. Bacteria provide essential metabolic functions, such as uric acid recycling, sulfate-reduction, acetogenesis, atmospheric nitrogen fixation and sustaining an anaerobic environment for protozoa, among other roles [3, 5, 11–14]. In addition to their vital contribution to cellulose digestion, gut symbionts are also a reservoir for proteins and lipids as termites digest microbes that get transferred via proctodeal trophallaxis from colony mates [15, 16].

Initially, three protozoa species and genera were described from the guts of *C*. *formosanus* workers [17–19]. A recent study employing a combination of single-cell PCR, microscopy, and 18S rRNA sequencing methods has confirmed that *C*. *formosanus* workers harbor three genera of protozoa; however, the number of species was increased to five [20].

The protozoa species in the guts of *C*. *formosanus* workers have functional niches. Members of the largest gut protozoa species, *Pseudotrichonympha grassii* (Trichonymphidae), consume wood particles via phagocytosis and degrade them [8, 21, 22], while the smaller protozoa further digest the metabolites and/or consume bacteria [9, 11, 18, 23].

In addition to the five protozoa species, at least 213 different bacterial species were identified in Formosan subterranean termite workers via clone-based 16S rRNA gene sequencing [8, 24, 25] and bacterial culture [26–28]. However, this number is probably underestimated compared to next-generation sequencing studies of specimens from closely related species [24, 29–31]. The bacterial symbionts are free-living in the gut lumen, attached to the gut wall, or associated with the protozoa as endo- or ectosymbionts [4, 32].

While extensive research has been conducted on eukaryotic and prokaryotic symbionts in the worker caste of termites, limited knowledge exists regarding symbionts associated with the reproductive caste. Given the importance of termite gut symbionts in colony nutrition, protozoa and/or bacteria are likely to play a crucial role in the establishment of new termite colonies.

Subterranean termite colonies are founded by a pair of winged reproductives (alates) during swarming season. These alates carry essential microbes acquired from their natal colony [33, 34] as the “starter package” of symbionts for the new colony, which is thought critical for successful colony initiation [35, 36]. During mass swarming events, hundreds of alates from different colonies aggregate in swarm clouds [37, 38]. After swarming, dealated males and females engage in tandem running to search for a suitable nesting site [39–41]. The close contact during tandem running allows them to investigate mechanical and/or chemical cues to assess potential partners [42–44]. Finding a high-quality partner is crucial for successful colony foundation since termites mate for life and the first generation of offspring is dependent on the nutritional resources of the founder pair [44, 45], consisting of proteins and lipids contained in the tissues of the dealate body themselves and in their gut protozoa and bacteria. During the biparental phase of the incipient colony, the founder pairs metabolize their own fat and protein reserves while digesting part of their symbiont population to sustain themselves and the first generation of offspring [34, 46]. Once the colony transitions to the alloparental phase, the founders inoculate the first brood of workers with the necessary symbionts to enable them to forage for and digest lignocellulose [46].

Numerous studies have investigated what factors impact the successful survival and growth of incipient colonies, including environmental conditions [46–50] and contributions by the colony founders. The latter largely comprises nutritional provisioning by the founding pair estimated from fat reserves [33, 51], dealate weights [49], parental nitrogen transfer [52], and from changes in the numbers of protozoa used to sustain the pair and the first larvae [53]. In contrast to the straightforward contributions of parental nutritional resources, levels of inbreeding play a rather complex role in the growth of incipient colonies in various subterranean termite species and its impact depends on other factors, such as colony age and cuticular microbial load [54–56]. Surprisingly, few studies have investigated the role of microbiota in incipient colonies. Cuticular bacterial and fungal loads causing pathogen stress impacted incipient colony survival of subterranean termites [56, 57]. Additionally, studies on the dynamic changes in protozoa numbers within the guts of colony founders of various subterranean termite species have confirmed the importance of gut symbionts during the early stages of an incipient colony and the transition from biparental to alloparental care [35, 53, 58, 59]. These gut protozoa serve as immediately available nutritional resource to obtain proteins and lipids for founder pairs, and as inoculum for future worker generations to confer the ability to efficiently digest lignocellulose [46].

Given the important dual role of the microbial symbionts in incipient colony nutrition, it is surprising that the potential impact of gut symbionts on pair formation of dealates at the earliest stage of colony initiation has not yet been explored. So far, it has been shown that size, weight and genetic diversity of dealates play a role in pair formation via competitive advantage and/or mate choice, depending on the species of termite [41, 45, 60], while kin selection and inbreeding avoidance is unlikely to influence pair formation [41, 56].

In this study, we hypothesized that the founder pair carries a core microbiota consisting not only of the obligate protozoan symbionts, but also essential bacteria, and that pair formation is influenced by the body weight as well as by the composition of protozoa and/or bacteria. In contrast to previous studies that used tandem running pairs, which might still change partners, we investigated pairs that had sealed themselves in incipient nest chambers and, thus, represented the final founder pair. We determined weight and protozoa numbers and described the diversity of bacterial taxa in the guts of *C*. *formosanus* founder pairs and dealates that remained single using 16S rRNA gene sequencing to test whether there is a difference in weight, protozoa numbers and/or bacterial diversity between paired and unpaired and male and female dealates from different populations.

## Material and methods

### Termite alates collection

Four populations of *Coptotermes formosanus* alates were collected in May 2021 from different locations in Louisiana separated by at least 4 km, which exceeds the swarming distance [38, 61]. The four populations were from Baton Rouge-Bluebonnet Swamp (BB), Baton Rouge-Botanic Garden (BG), New Orleans (NO), and St. Gabriel (SG). Exact location and dates of collection are given in S1 File. The light traps for alate collection were set up on the ground in open areas within one hour after sunset. Each light trap consisted of an 18.9L plastic bucket with a bright neon ring light to attract swarming alates. The bucket was placed on a white mat (1.5 x 1.5 meters in size) to collect additional alates that landed outside of the bucket (Fig S1 in S3 File). Corrugated cardboard was cut into small pieces (8 x 8 cm) and placed on the white mat and inside the bucket as artificial nest sites (Fig S1 in S3 File). After the swarm event, alates were carefully collected by wrapping all the pieces of corrugated cardboard with the mat, placing the bundle into the bucket, closing the lid and immediately transporting the alates to the laboratory. In the lab, the contents of the bucket were transferred to a large container box of 65 x 50 x 25 cm (length by width by height) where during the night alates continued to shed their wings, formed tandem pairs, and moved into the provided cardboard nest sites. The following day, pairs of dealates that were found making a nest chamber in the corrugated cardboard were collected and classified as “paired” (Fig S1 in S3 File). Dealates that remained single and did not crawl into a nest site but kept running throughout and along the edge of the container were labeled as “unpaired”. Nest sites were provided in excess and were not a limiting factor for pair formation. Tandem running dealates were not included in this study. Dealates were sexed by examining the terminal abdominal sterna with a stereomicroscope [62].

### Dealate weights and protozoa counts

Eighty dealates were selected for the study consisting of twenty paired and unpaired male and female dealates from the four populations (S1 File). Prior to dissection, each individual dealate was weighted. The hindgut of each dealate was then extracted with micro-scissors and forceps and pierced to release the gut content and flushed with 1000 μl PBS buffer. Number of total protozoa and of the protozoa species *Pseudotrichonympha grassii*, were counted in 10 μl of the solution using a Leica DM750 microscope. Protozoa counts were replicated three times and multiplied by 100 to determine the protozoa number per gut. The percentage of *P*. *grassii* (number of *P*. *grassii* / total number of protozoa in the individual sample) was also calculated. Protozoa numbers of the population BG were counted before the decision was made to count *P*. *grassii* separately; therefore, no *P*. *grassii* numbers or percentages were available for this population. Following confirmation of normal distribution of the data we assessed the effects of pairing status, sex of the dealates, and population on weight, total protozoa number, *P*. *grassii* number and percentage of *P*. *grassii*. We first performed univariate tests, i.e., two-tailed, unpaired t-tests with Welch’s correction (modified Student’s t-tests for unequal variances) for the factors pairing status and sex, and a one-way ANOVA for the population factor significances. Interactions of the factors were further tested with multivariate analysis of variance (MANOVA). A factor was only considered to be significant when both MANOVA and univariate tests showed significance (P < 0.05). Box-and-whisker plots were created using GraphPad Prism version 8.0.0 (GraphPad Software, San Diego, CA). To determine whether there was a correlation between body weight and protozoa numbers, we conducted Spearman correlation tests in R [63]. The Spearman correlation tests yielded a correlation coefficient (rho) indicating the strength of the correlation and were considered significant if the P-value was less than 0.05.

### Gut content DNA extraction

The remaining gut content was homogenized with a sterile pestle and total DNA was extracted using the Dneasy Blood and Tissue kit (Qiagen, Valencia, CA, United States). The concentration of extracted DNA was confirmed using an Invitrogen Qubit 4 Fluorometer (Thermo Fisher Scientific, Wilmington, DE) with the Qubit dsDNA BR Assay kit (Invitrogen^™^, life technologies^™^, CA). Aliquots of 10 μl containing 25 ng of extracted DNA from each sample were sent to University of New Hampshire Hubbard Center for Genome Studies for next-generation sequencing.

### Illumina sequencing

The DNA samples were processed for sequencing at the University of New Hampshire Hubbard Center for Genome Studies. To capture a broad range of bacterial biodiversity, the V4-V5 hyper variable region of the 16S rRNA gene was amplified using the universal 16S rRNA primer set (515F and 926R) as described in previous studies [64]. The amplified PCR products were sequenced on the Illumina NovaSeq platform using the 2x250 bp paired end sequencing protocol, following the Illumina Nextera Dilute library preparation protocol (Illumina, San Diego, CA).

### Quality control and generation of Amplicon Sequence Variants

Bioinformatics was performed using QIIME2 (version 2022.2) [65], following the pipeline described by Estaki et al. [66]. The demultiplexed sequencing reads were obtained in FASTQ format after Illumina sequencing. The demux plugin in QIIME2 was used to visualize the demultiplexed sequences before beginning the downstream analysis. Sequence ends with low Phred quality scores (< 30) were trimmed using the DADA2 plugin in QIIME2 [67]. Several samples that had low sequencing depth were re-sequenced. Sequences from the same sample that were generated twice were merged. Only forward sequences were used for the subsequent analyses because the quality of the reverse sequences was generally poor. Approximately 3.1 million reads across all samples representing 1,489 Amplicon Sequence Variants (ASVs), which are bacterial sequences that differ by at least one base pair, were obtained as a result of the DADA2 procedure. All raw sequence data used in this study are accessible on the NCBI database under BioProject PRJNA950480.

### Rarefaction

The total sequence depth (number of reads) usually varies widely between samples. In order to test for sufficient sequencing depth and compare samples at the same sequencing depth for diversity analysis, alpha rarefaction implemented in QIIME2 was used to subsample sequence reads without replacement to the common sequencing depth that was equal to the sample with the lowest sequencing depth [66]. The qiime diversity plugin’s alpha rarefaction methods were used to plot alpha rarefaction curves showing the relationship between alpha diversity and sequencing depth. Three different alpha diversity indices were used: The ASV richness index measures number of ASVs, Faith’s PD index calculates the phylogenetic distance between the ASVs [68], and the Shannon diversity index scales the richness of ASVs based on their evenness [69]. In addition, sample size- and coverage-based rarefaction curves were generated utilizing the R package iNEXT (iNterpolation/EXTrapolation), as described in Hsieh et al. [70].

### Taxonomical assignment

The ASVs were taxonomically classified by comparing them to bacterial 16S rRNA sequences in the SILVA 138 database using BLAST [71]. All eukaryotic ASVs belonging to termite host and protozoa were excluded. Sequences with <97% similarity to database references were classified as unassigned sequences. Sequences were aligned multiple times using the MAFFT approach, and the highly variable positions of the alignments were filtered out using the mask command [72]. A midpoint-rooted phylogenetic tree was constructed using these aligned and masked sequences [73]. Taxonomy barplots were generated showing the relative abundances of taxa at different taxonomic levels. To further validate the taxonomic assignment of certain key bacteria, we conducted a BLAST search against the NCBI database, ensuring rigorous confirmation of the bacterial ASVs.

### Alpha and beta diversity

To evaluate alpha diversity (bacterial diversity within dealate samples) of all bacterial ASVs, both incidence-based measures (ASV richness and Faith’s PD), and abundance-based indices (Pielou’s evenness and Shannon diversity) were employed. The ASV richness metric quantifies the number of distinct taxa or genetic variants present in a sample and provides a measure of taxonomic diversity [74]. Faith’s PD metric takes into account the phylogenetic relatedness of the ASVs and provides a measure of evolutionary diversity [68]. In addition, the Shannon diversity metric, which measures predictability of the species identity of any bacteria drawn at random based on richness and evenness of ASVs [69], and Pielou’s evenness, which divides the Shannon index by its maximum possible value under equal distribution [75] were also used to compare diversity across samples. The QIIME2 “qiime diversity” plugin was utilized to perform Kruskal-Wallis ANOVAs with Benjamini-Hochberg correction to test for significant effects of pairing status, sex, and population on bacterial alpha diversity of dealates. Total protozoa and *P*. *grassii* numbers and percentages were correlated to bacterial ASV richness (number of ASVs) and abundances based on the rarefied number of sequence reads of ASVs using the Spearman’s rank correlation test in R (version 4.2.2). Similarly, the number of rarefied reads of highly abundant bacteria in our dataset that were identified by previous studies as putative protozoa symbionts were correlated to protozoa numbers to test for associations.

Beta diversity (bacterial diversity among dealates with different pairing status, sex or population) was evaluated using four indices for creating distance matrices consisting of two incidence-based measures, Jaccard [76] and Unweighted Unifrac [77], and two abundance-based measures, Bray-Curtis [78] and Weighted Unifrac [79]. The Unifrac indices take into consideration the phylogenetic distances between the ASVs to determine microbiota differentiation, whereas the Jaccard and Bray-Curtis indices compute the distance matrices without considering phylogenetic relationships. ADONIS, a multifactorial Permutational Multivariate Analysis of Variance (PERMANOVA) procedure in QIIME2 [80], was used to test for significant differentiation among dealates based on pairing status, sex and population origin. Pairwise PERMANOVA tests with 999 permutations were used to confirm significance. The study also used Permutational Analysis of Multivariate Dispersions (PERMDISP) with 1,000 permutations to test the homogeneity of each factor’s multivariate dispersion and to ensure that the assumptions of the ADONIS test were not violated.

## Results

The raw data, including information on termite dealates such as weight, total protozoa number, number of *P*. *grassii*, and the percentage of *P*. *grassii*, is available in S1 File.

### Dealates’ body weight

**Fig 1:**
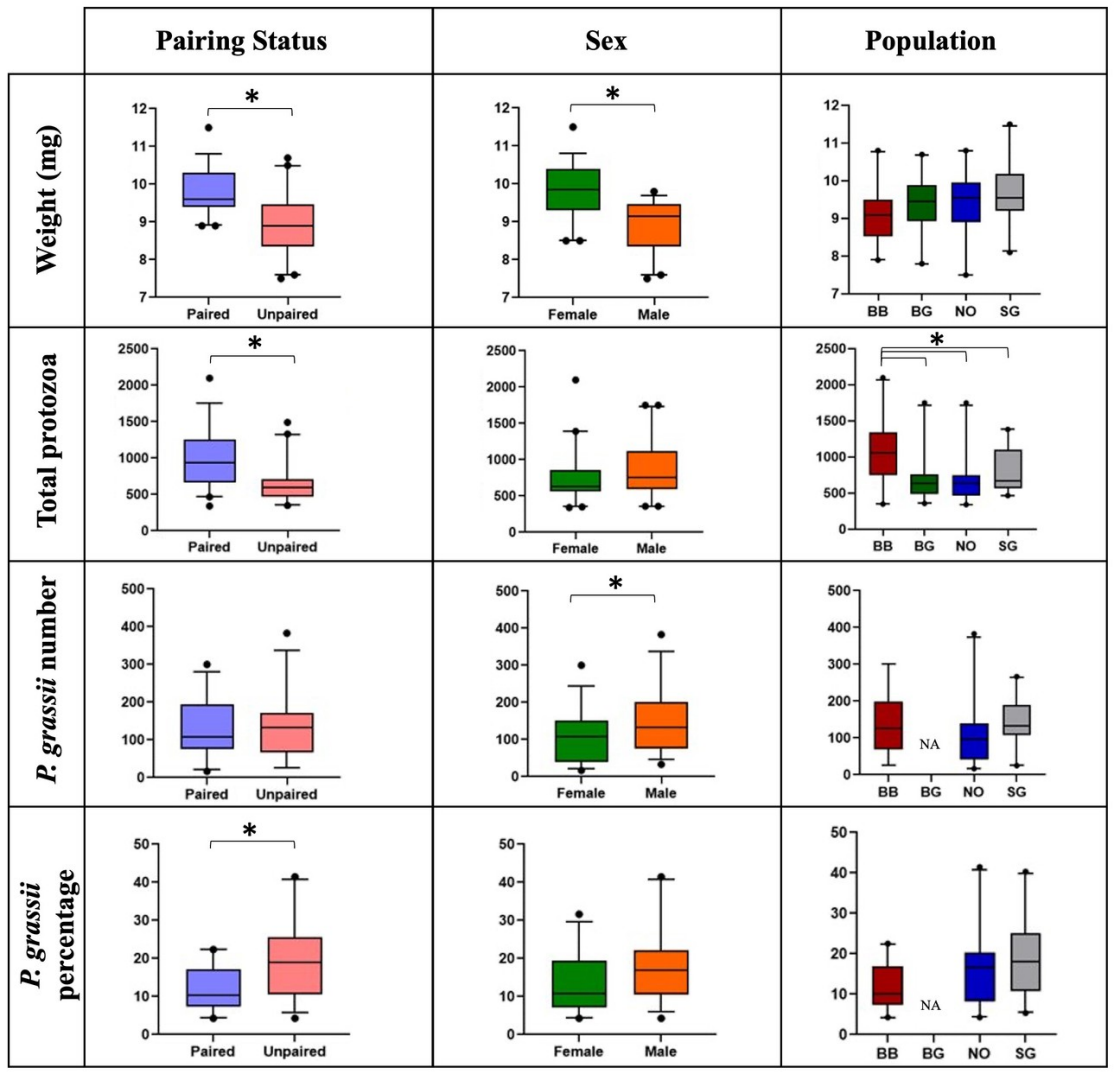
Dealates’ weight, number of total protozoa, *P*. *grassii* and percentage of *P*. *grassii* separated by pairing status, sex, and population. Box plots show the median with quartiles, 5^th^ and 95^th^ percentiles (whiskers) and extreme values for the minimum and maximum of the protozoa counts in each group. Significant results (P < 0.05, Welch’s t-test or ANOVA confirmed by MANOVA) are marked by asterisks (*); *P*. *grassii* counts from the BG population were not available (NA).

Body weight varied significantly with pairing status and sex of the dealates. Paired dealates and females had higher body weights (P < 0.0001 for both Welch’s t-test and MANOVA, n = 80, Table S1 in S4 File). However, the lack of correlation between body weights of dealate partners showed that heavier females did not pair with heavier males (P = 0.975, Spearman’s rank correlation, Table S2 in S4 File). No significant differences in body weights were found among populations (Fig 1, Table S1 in S4 File).

### Protozoa counts

The three protozoa genera, *Pseudotrichonympa*, *Holomastigotoides* and *Cononympha* were morphologically identified in all dealate samples. Paired dealates had significantly higher numbers of total protozoa than single dealates (P < 0.0001 for both Welch’s t-test and MANOVA, n = 80, Fig 1, Table S1 in S4 File). Although the number of *P*. *grassii* did not show significant difference, the percentage of *P*. *grassii* was significantly higher in unpaired than paired dealates (P ≤ 0.0004 for both Welch’s t-test and MANOVA, n = 60). The significant interaction between pairing status and population (P = 0.025, MANOVA, Table S1 in S4 File) indicated that this effect of pairing status on *P*. *grassii* percentage was dependent on population and mainly caused by significant differences between paired and unpaired dealates in NO (P = 0.0328) and SG (P = 0.0005) populations but not in the BB population (P = 0.7423). Number of total protozoa, *P*. *grassii* and percentage of *P*. *grassii* were not correlated between paired males and their female partner (P > 0.1, Spearman’s rank tests, Table S2 in S4 File).

Male dealates had significantly higher *P*. *grassii* numbers than females (P = 0.017, Welch’s t-test and P = 0.021, MANOVA n = 60). Although males were found to carry more total protozoa than females (mean number of total protozoa in males was 870.9 compared to 749.9 in females, Table S1 in S4 File), the difference in total protozoa numbers was not significant due to high variability among individuals (P = 0.138, Welch’s t-test and P = 0.065, MANOVA, n = 80) (Table S1 in S4 File). Conservatively, we did not consider the difference in the percentage of *P*. *grassii* relative to total protozoa numbers between sexes as significant, because the Welch-test showed only marginal difference (P = 0.069) as opposed to MANOVA (P = 0.032).

Dealates from different population origins showed significant difference in total protozoa numbers (P = 0.0073 (BB vs NO); P = 0.0093 (BB vs BG); P = 0.035 (BB vs SG)), but not in *P*. *grassii* numbers or percentages (Fig 1, Table S1 in S4 File). The MANOVA tests showed that there were no significant interaction effects, except for the pairing status and population interaction for the percentage of *P*. *grassii* mentioned above (Table S1 in S4 File).

### Correlation test between dealate weights and protozoa counts

No significant correlation was found between the weight of dealates and any of the protozoa counts, which included total protozoa, *P*. *grassii* counts, and percentage of *P*. *grassii* (P > 0.062, Spearman’s rank correlation, Table S2 in S4 File). Thus, weight did not confound protozoa counts. However, significant positive correlations were observed between the total protozoa count with *P*. *grassii* counts (P = 0.001, as well as between *P*. *grassii* counts and the percentage of *P*. *grassii* (P ≪ 0.0001, Rho = 0.765, Table S2 in S4 File). These correlations were expected, as the total protozoa count includes protozoa *P*. *grassii*.

### Sequencing depth-, sample- and coverage-based rarefaction

A total of 4,218,515 raw sequence reads were generated across all 80 samples. After strict quality control, 3,106,265 reads representing a total of 1,489 ASVs ranging from 16–212 per sample were obtained. The rarefaction curves for ASV numbers, Faith’s PD and Shannon diversity plateaued for all samples at a sequencing depth of less than the minimum sequencing depth of 892, indicating that sufficient sequencing depth was achieved to represent ASV richness and diversity present in each sample (Fig S2 in S3 File).

The sample-based rarefaction curves for the effective diversity calculated based on ASV richness for all dealate samples combined began to level off slightly at the actual sample size (n = 80), but the extrapolated portions of the richness curves continued to increase with additional samples. However, the curves for the effective diversity of the Shannon and Simpson inverse indices only minimally increased with doubling the sample size, which indicates that all common ASVs within the dealates’ gut bacterial community were collected (Fig S3A in S3 File). The coverage-based rarefaction curves revealed that 80 samples achieved over 95% sample coverage. Doubling the sample size increased sample coverage only slightly to 98% (Fig S3B in S3 File). These rarefaction approaches confirmed that sequencing and sampling effort was sufficient to capture the majority of bacteria and their diversity.

### Taxa composition

Of the 1,489 total ASVs (3,106,265 reads) generated in this study, 624 ASVs (298,864 reads) were bacteria that matched to references in SILVA with ≥ 97% similarity. The rest of the ASVs were filtered out because they were either assigned to Eukaryotes (228 ASVs with 343,200 reads) or remained unassigned (605 ASVs with 113,583 reads) due to the absence of close references in the SILVA database.

The 624 identified bacterial ASVs belonged to 19 bacterial phyla across all dealate gut samples (S2 File). The top six most abundant phyla (>1% relative abundance) were Bacteroidota (50.28%, with 80 ASVs), Proteobacteria (21.21%, with 108 ASVs), Firmicutes (13.06%, with 101 ASVs), Spirochaetota (11.42%, with 218 ASVs), Desulfobacterota (1.41%, with 19 ASVs) and Actinobacteriota (1.07%, with 41 ASVs). These six phyla represented over 98.43% of identified bacterial sequences and 91.03% of the identified bacterial ASVs (Table S3 in S4 File).

**Fig 2:**
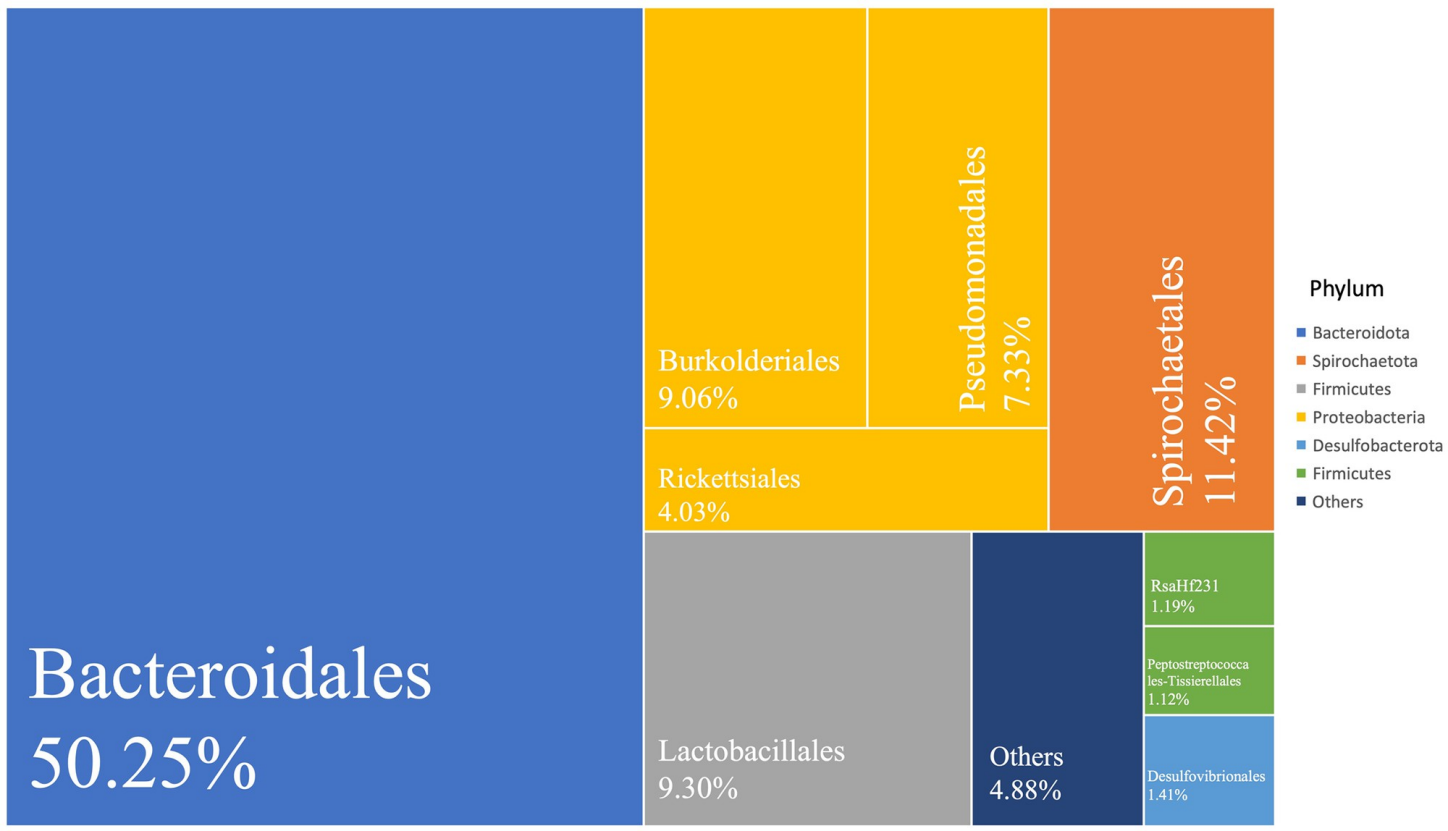
Summary of the dominant orders (>1% relative read abundance) in *C*. *formosanus* dealates. The abundance of the top 9 orders along with the remaining bacteria combined under Others is represented by the area of each rectangle, which is proportional to the percentage of bacterial reads.

At the order level, a total of 67 bacterial orders were identified. Bacteroidales was the most dominant order, accounting for 50.25% of the total bacterial abundance, followed by Spirochaetales (11.42%), Lactobacillales (9.30%), Burkholderiales (9.06%), Pseudomonadales (7.33%), Rickettsiales (4.03%), Desulfovibrionales (1.41%), Peptostreptococcales-Tissierellales (1.12%) and RsaHf231 (1.19%) of the Firmicutes. These top 9 orders, each with > 1% abundance, contained over 95% of identified bacterial reads (Fig 2).

**Fig 3:**
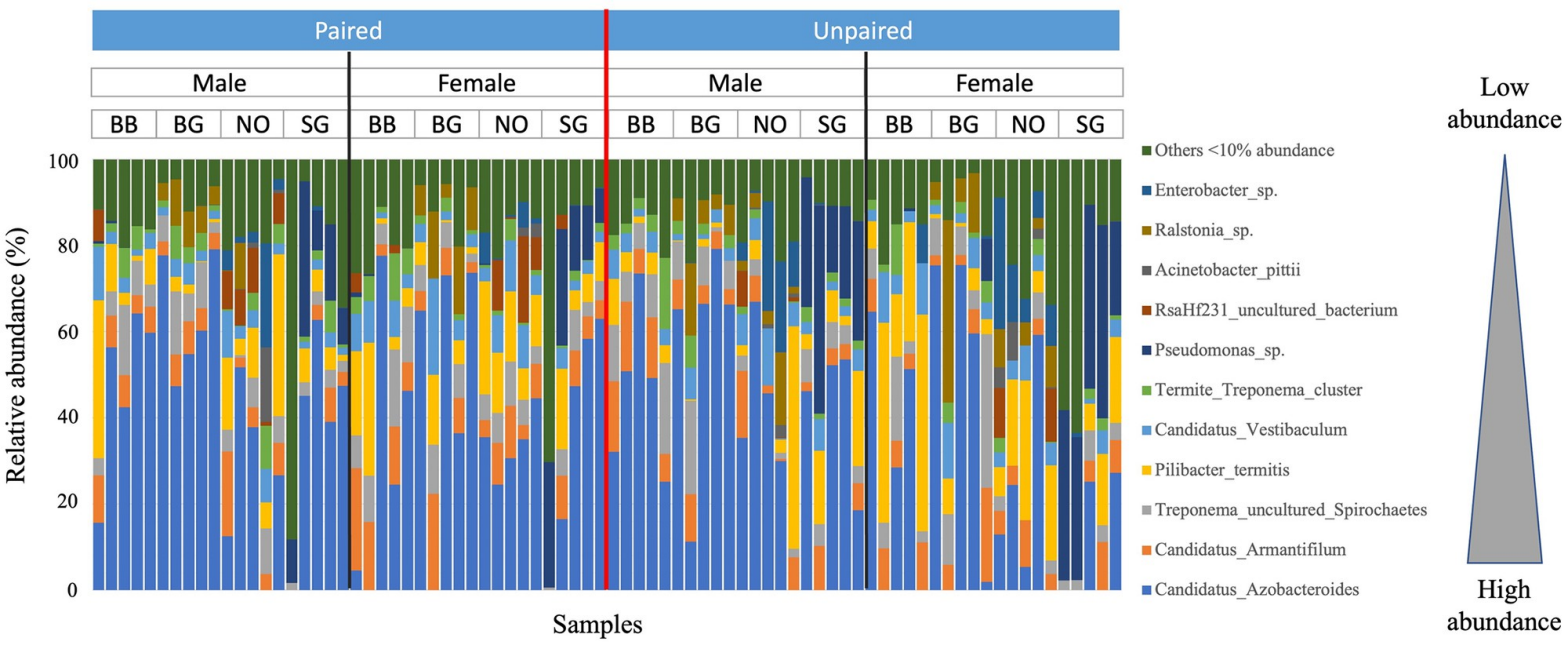
Relative abundances of the most dominant bacterial ASVs. Samples are ordered by pairing status (Paired, Unpaired), sex (Male, Female) and population (BB, BG, NO, SG). Bacterial ASVs with less than 10% relative abundance were combined into “Others <10% abundance”.

The top-ranked ASVs, each with an abundance of over 10% in at least one sample, ordered by overall abundance from high to low across all samples were: *Candidatus* Azobacteroides, *Candidatus* Armantifilum, Treponema uncultured Spirochaetes, *Pilibacter termitis*, *Candidatus* Vestibaculum, Termite Treponema cluster, *Pseudomonas* sp., RsaHf231 uncultured bacterium, *Acinetobacter pittii*, *Ralstonia* sp., and *Enterobacter* sp. (Fig 3).

The most abundant genus, *Candidatus* Azobacteroides, accounted for up to 79.2% of bacterial abundance per sample, although not all dealates carried it. Ten females and four males across all populations (17.5% of a total of 80 dealates) did not have *Ca*. Azobacteroides. Almost twice the number of dealates without *Ca*. Azobacteroides were found among the unpaired dealates (7 females and 2 males) as compared to paired samples. In almost all pairs, both partners (in 36 pairs) or at least one of the partners (in 3 pairs) carried *Ca*. Azobacteroides. There was only one pair of dealates from the SG population where both partners lacked *Ca*. Azobacteroides. The abundance of *Ca*. Azobacteroides was positively correlated with the numbers of its host protozoa *P*. *grassii* (P = 0.0004, Rho = 0.4391, n = 60, Spearman’s rank correlation), and also with total protozoa number (P = 0.0135, Rho = 0.2752, n = 80, Spearman’s rank correlation).

We further tested if there is a correlation between protozoa number and the abundance of other dominant bacteria previously identified as putative protozoa symbionts (Table S4 in S4 File). While *Ca*. Armantifilum numbers were not correlated with *P*. *grassii* numbers (P = 0.2075, Rho = 0.1651, Spearman’s rank correlation test), its abundance was correlated to total protozoa number (P = 0.0367, Rho = 0.2340, Spearman’s rank correlation test); however, *Ca*. Vestibaculum abundance was not correlated to either measure of protozoa abundance (P > 0.2260) indicating that this ASV was not associated with protozoa.

While BLAST analysis against NCBI reference database confirmed most SILVA taxonomic assignments, our *Ca*. Armantifilum and *Ca*. Vestibaculum ASV sequences differed substantially (89–91 percent identity) from the *Ca*. Armantifilum (Acc. No. FN377751-FN377758) and *Ca*. Vestibaculum (AY540335) reference sequences of NCBI; however, they matched to several termite-related uncultured Bacteroidetes bacteria. For example, the *Ca*. Armantifilum ASVs matched with ≥98% to GQ502508.1 and GQ502483.1 in *C*. *formosanus* and KP690878.1 in *C*. *curvignathus*; the *Ca*. Vestibaculum ASVs matched 100% to GQ502489.1 and 98% to GQ502490.1 sequences of *C*. *formosanus* in the NCBI database.

Unlike the termite-specific bacterial symbionts, including the candidate genera, *Treponema* spirochetes and *P*. *termitis*, environmental bacteria, e.g., *Pseudomonas*, *Ralstonia* and *Enterobacter* species, were less abundant overall and largely restricted to specific populations and individual samples.

### Alpha-diversity of dealates’ gut bacterial community

Pairing status had no significant effect on the bacterial richness, diversity, or phylogenetic distance among ASVs of the dealate samples (P > 0.240, Kruskal-Wallis test for all indices, Table S5 in S4 File) nor was bacterial diversity correlated between partners (P > 0.161, Spearman’s rank correlation for all diversity indices). Interestingly, male dealates had significantly higher gut bacteria richness (ASV numbers, P = 0.048, Kruskal-Wallis test) and marginally higher phylogenetic diversity (Faith’s PD, P = 0.061, Kruskal-Wallis test) compared to the female dealates. The population factor showed a significant effect on bacterial diversity only when the phylogenetic distance was taken into account (Faith’s PD, P = 0.019, Kruskal-Wallis test), but not when only the number of bacteria taxa (ASV numbers, P = 0.156, Kruskal-Wallis test) was considered (Table S5 in S4 File). Analysis of evenness and Shannon diversity metrics revealed no statistically significant differences for all three factors.

Alpha-diversity correlation tests showed that number of ASV reads, i.e., abundance, was significantly correlated with total protozoa number (P = 0.049, Rho = 0.221) and number of ASVs, i.e., bacteria richness, was significantly correlated to *P*. *grassii* numbers (P = 0.042, Rho = 0.264, Spearman’s rank correlation), but both ASV metrics were not correlated to dealates’ weight and percentage of *P*. *grassii*. Moreover, Shannon diversity, Pielou’s evenness and Faith’s PD of bacteria communities were not correlated to any of the protozoa counts or delalate weight (Table S6 in S4 File).

### Beta-diversity of bacterial community of dealates

Both multiple-factor ADONIS analysis (Table S7 in S4 File) and single-factor PERMANOVA with Benjamin-Hochberg correction (Table S8 in S4 File) that employed four different diversity indices (Weighted UniFrac, Bray-Curtis, Unweighted UniFrac and Jaccard) indicated that the pairing status of dealates did not affect their bacterial gut community. Additionally, the dispersion of bacterial communities between paired and unpaired dealates did not differ significantly (P > 0.469, PERMDISP with Benjamin-Hochberg correction, Table S8 in S4 File), suggesting that the assumptions underlying the ADONIS test were not violated.

Bacterial composition between male and female delates showed a significant difference when examining solely the presence of ASVs without regard to phylogenetic distance (P = 0.008 for Jaccard index) and marginal differentiation when phylogenetic distance was taken into account (P = 0.055 for Unweighted UniFrac, ADONIS); however, this difference was not observed when considering the relative abundance of bacteria (P ≥ 0.294 for Bray-Curtis and Weighted Unifrac indices) (Tables S7 and S8 in S4 File). Despite the significant and marginal differences, sex explained only a minute portion of the variance in bacterial community differentiation (R2 = 0.018 and 0.020 for Jaccard and Unweighted Unifrac, respectively, Table S7 in S4 File). There was significant interaction of sex of dealates and pairing status for the incidence-based indices (P = 0.012 for Unweighted Unifrac and P = 0.013 for Jaccard, ADONIS), but, again, this explained less than 3% of the variance (Table S7 in S4 File). Homogeneity of dispersion confirmed that the underlying assumptions for the ADONIS test were not violated (P > 0.648 for all indices, PERMDISP, Table S8 in S4 File).

The gut bacterial composition varied significantly among populations according to all four beta diversity indices (P ≤ 0.006, ADONIS, Table S7 in S4 File). Of the three factors, population explained the largest amount of the variance among microbiota, i.e., 7.6% to 14.4% across the different indices. However, we also detected significant differences in dispersion (P < 0.05, PERMDISP, Table S8 in S4 File) in three of the four beta diversity metrics, which makes the interpretation of the ADONIS results for the population factor difficult, since they are confounded by non-homogeneous dispersion. Population-related differences in bacterial communities are probably a reflection of differences in environmental bacteria, which is expected since different locations have different bacterial profiles in soil and wooden food sources.

## Discussion

The short- and long-term success of incipient termite colonies depends on the resources of the founder pairs and their life-long commitment to their partners, suggesting that some form of selection among dealates should exist depending on the alate’s quality [35, 49, 52, 56, 81–83]. The alates’ fat body together with gut microbiota are the foremost resources of nutrition for sustaining the royal pair and their first brood of larvae during the biparental phase of early colony establishment [49, 53]. Therefore, we tested if body weight, protozoa numbers and bacteria composition were associated with pair formation in *C*. *formosanus* dealates.

### Body weight influences pair formation

Our study showed that male and female dealates found in pairs in nest chambers were significantly heavier than those remaining single. While selection in termites for body size and weight is generally expected to be weak compared to their roach-like ancestors [84], studies have associated larger weights of male and female alates and higher combined weights of founder pairs to successful establishment and increased growth of incipient colonies in *C*. *gestroi* [49].

Despite arguments that founders do not require large metabolic reserves, for example, mature colonies are thought more likely to invest in small alates with increased dispersal ability [49], initial colony success and growth has been linked to the resources of the founder pair. Chouvenc’s [49] study demonstrated that larger individuals could provide more resources for raising larvae, as evidenced by the founder’s weight loss in *C*. *gestroi*. However, this weight advantage has not been tested for non-random pair formation in the field since that study was conducted under controlled laboratory conditions with artificial pairings. Other studies have also shown selection in termite imagoes for large and/or heavy partners, such as *Zootermopsis nevadensis* [45] and *Reticulitermes* spp. [60, 85]. In addition, size has been found to increase the likelihood of tandem running in *C*. *formosanus* dealates, with males in tandem pairs with females having significantly larger heads than single males, although there was no difference in weight between members of tandem pairs and single termites [41].

In contrast to the previous study by Husseneder and Simms [41], our present study showed that pairs in nest chambers were significantly heavier than single dealates. These discrepancies can be explained by the fact that tandem running is not necessarily the final partner choice, as opposed to creating a nest chamber together, and switches of tandem partners occur frequently, which likely added variation masking the differences in weight. Overall, it is not surprising that the weight of both sexes is a factor determining pair formation, as weight is an indicator for the amount of resources that a founder pair can invest in biparental care for a new colony [49, 85].

### Protozoa numbers are sex-biased and associated with pair formation

Similar to body weight, protozoa numbers have been suggested as a proxy for the nutritional resources available to termites, including lipids and proteins [33, 35]. In our study, we observed that male *C*. *formosanus* dealates harbored higher numbers of the largest protozoa species, *P*. *grassii*, than females, despite being smaller in body size and weight. This sex-based difference in protozoa numbers has also been observed in *Reticulitermes speratus* by Shimada et al. [35], who suggested that kings ingest more wood in the early stages of colony formation and may donate more nutrients to their partners and offspring than queens. Similar male-biased nutrition contributions have been observed in other termite species, including *Zootermopsis nevadensis* and *Hodotermopsis japonica* [82, 86], and have been attributed to offsetting the greater resource requirement for egg production [35, 53].

In this study, we found that a high total number of protozoa in both male and female dealates of *C*. *formosanus* increased the likelihood of pair formation and establishment of nest chambers, independent of, i.e., not correlated to weight. This is not surprising since the gut protozoa, which are obtained via proctodeal trophallaxis from their natal colony prior to the dispersal flight, serve as a nutrition source during colony initiation [35]. However, similar to our observations regarding weight, protozoa numbers in male and female partners were not correlated with each other, indicating that the prevalence of dealates with high numbers of protozoa among pairs is likely not the result of a stringent process of mutual choice.

The absolute number of *P*. *grassii* was not significantly different between paired and single dealates and the ratio of *P*. *grassii* relative to total protozoa counts was significantly lower in paired dealates. This finding may appear counterintuitive since *P*. *grassii* is the largest protozoa species and is crucial for breaking down wood particles for smaller protozoa species to complete digestion [87, 88]. However, at this early stage of colony development, only limited amounts of wood surrounding the nest chamber are digested by founder pairs to meet their carbohydrate requirements and that of the first larvae [35]. The large protozoa of the genus *Pseudotrichonympha* are mostly lost prior to the imaginal molt of *C*. *formosanus* alates [36] and have to be re-acquired from workers, which explains the considerable variation in the protozoa content of dealates [89]. *Pseudotrichonympha* is also the least abundant genus by cell number in the hindgut of *C*. *formosanus* and *C*. *gestroi* workers [20]. Therefore, it is likely that the biomass supplied by the smaller species of protozoa, i.e., *Holomastigotoides hartmanni*, *Holomastigotoides minor*, *Cononympha leidyi and Cononympha koidzumii* [20], is of greater importance in pair formation and colony initiation than high numbers of large *Pseudotrichonympha*. However, protozoa abundances increase dramatically in kings and queens before the emergence of the first worker generation in preparation for vertical transmission of the symbiont inoculum to the worker caste [35, 58].

Since these protozoa are obligate symbionts and cannot be obtained from the environment, it is important that core protozoa species are present at least in one of the partners and are preserved throughout the biparental phase to initiate the alloparental phase by inoculating the first workers [33]. In our study each dealate carried all three genera of protozoa; however, we did not identify protozoa to the species-level. A recent study showed that alates in both *C*. *formosanus* and *C*. *gestroi* do not necessarily carry all protozoa species. Nevertheless, there is a high probability that biparental transmission of the combined protozoa communities of both parents will supply the colony with all three protozoa genera and at least 4–5 species [89]. Moreover, there seems to be some functional redundancy among protozoa species, since over half of *C*. *formosanus* colonies harbored only 4 out of the 5 species [89].

Further studies are needed to describe the roles of the different species of protozoa in short-term and long-term growth of incipient colonies and how protozoa shape the bacteria community. In our study total protozoa number was significantly correlated to bacterial abundance and *P*. *grassii* protozoa counts were significantly correlated with bacterial richness, indicating that protozoa may play a role in shaping the bacterial community by providing niches and metabolites for bacteria species.

### Most dominant bacteria in dealates are termite-specific obligate core bacteria

The gut bacteria of workers of *Coptotermes* species have been extensively studied in previous research [5, 13, 24, 25, 30]. However, this is the first study to describe the bacterial diversity present in the guts of *C*. *formosanus* dealates and to investigate the links between microbiota and pair formation as well as the relationship between protozoa abundance and bacterial diversity in this termite species.

The dominant bacteria groups found in dealate samples generally reflect the core phyla including Bacteroidota, Proteobacteria, Firmicutes, and Spirochaetota as well as the most dominant orders and genera found in the guts of *C*. *formosanus* workers [13, 24, 25, 28]. For example, *Ca*. Azobacteroides and ASVs assigned to *Ca*. Armantifilum of the order Bacteroidales were among the top 5 most abundant taxa in this study. *Candidatus* Azobacteroides, an obligate endosymbiont of *Pseudotrichonympha* protozoa species confirmed by the strong correlation of *Ca*. Azobacteroides and *P*. *grassii* numbers in our study, has been found to be dominant in *C*. *formosanus* termites across the globe regardless of caste [59, 90–93]. Previous studies have demonstrated that *Ca*. Azobacteroides is involved in dinitrogen fixation and provides amino acids and cofactors to the host [8, 94]. Interestingly, not all dealates harbored *Ca*. Azobacteroides indicating imperfect vertical transmission from the natal colony to the swarming alates as described for their protozoa [89]. However, similar to Velenovsky et al.’s [89] study, biparental transmission ensures that almost all incipient colonies receive the necessary inoculum; only one pair of dealates did not contain this vital symbiont, while *Ca*. Azobacteroides was supplied in 36 pairs by both parents and in three pairs by one parent. A study is currently investigating the importance of *Ca*. Azobacteroides abundance in early colony development.

*Candidatus* Armantifilum was originally described as an ectosymbiont of the protozoa *Devescovina* spp. [95] and has been reported among the dominant gut bacteria of workers in a diverse range of termite genera including *Coptotermes*, *Heterotermes*, *Reticulitermes*, *Neotermes*, and *Cryptotermes* [95–97]. Bacteria with similarity to the genus *Ca*. Vestibaculum, an ectosymbiont of protozoa *Staurojoenina* in termites of the family Kalotermitidae [98, 99] were also among the dominant gut bacteria in *C*. *formosanus* dealates. As these ectosymbionts are still uncultured and their genomes not yet sequenced, their roles can only be inferred from similar bacterial symbionts of protozoa. These putative roles include providing essential amino acids and cofactors to protozoa hosts, nitrogen fixation [94], consumption of hydrogen produced by the protozoa [100], protection of protozoa from oxygen diffusing into the gut [101, 102] and maintenance of the cytoskeletal structure of their host flagellate [103]. Interestingly, *C*. *formosanus* members do not possess protozoa of the genera *Devescovina* or *Staurojoenina*. Although the ASVs in our study were taxonomically assigned to the candidate genera Armantifilum and Vestibaculum by SILVA, the low sequence identities to *Ca*. Armantifilum and *Ca*. Vestibaculum references in NCBI GenBank suggest that the corresponding ASVs in *C*. *formosanus* specimens were not the same species as originally described. However, both of these ASVs showed uncultured Bacteroidetes found in previous studies in *C*. *formosanus* [25] and *C*. *curvignathus* [104] as top matches in NCBI. Thus, both bacteria species, although not well described, belong to the core bacteria of *Coptotermes* species. In our study, the abundance of ASVs assigned as *Ca*. Armantifilum was correlated to total protozoa number, but not *P*. *grassii* abundance, suggesting an association with *Cononympha* or *Holomastigotoides*, but not *Pseudotrichonympha* species in *C*. *formosanus* termites. In contrast, numbers of ASVs assigned to *Ca*. Vestibaculum showed no correlation and, thus, no association with protozoa in *C*. *formosanus* dealates.

Other dominant bacteria in dealates, such as the genera *Treponema* (Spirochaetales) and *Pilibacter* (Lactobacillales) have been previously recognized as dominant bacteria in the worker caste [24, 25, 28, 105]. Treponema ASVs were highly abundant in dealates, which is expected since Spirochaetota were the second most dominant phylum in *C*. *formosanus* workers previously collected from Louisiana [25] and *Treponema* is the most abundant spirochete genus in subterranean termite guts [106]. Members of the genus *Treponema* are known for H_2_-CO_2_ acetogenesis [107] and nitrogen fixation [108]. The fermentation process of *Treponema* is thought to support cellulose digestion in protozoa [14].

*Pilibacter termitis* (Lactobacillales), a species cultured and described from *C*. *formosanus* workers [109] was highly prevalent in dealates. Dominant uncultured clones in *C*. *formosanus* worker guts [24, 25] were retroactively identified as *Pilibacter* species by sequence identity [28, 109] after the official description of this species [109]. Furthermore, *Pilibacter* and *Treponema* species were reported at high abundance as free-living bacteria in *C*. *gestroi* workers [30].

The presence of core bacteria in dealates and their similarity to the worker caste is expected for two reasons. Firstly, alates obtain their symbionts, i.e., protozoa and bacteria, after their molt to imago from the workers of the natal colony via proctodeal trophallaxis [33, 34]. Secondly, colony founders have to contain the entire “starter package” of obligate and supporting symbionts for inoculating the first brood of workers since most obligate symbionts cannot be obtained from the environment.

### Bacteria diversity was sex-biased but not associated with pair formation

The significantly higher bacterial richness, marginally higher phylogenetic diversity in male dealates and differentiation in beta diversity between the sexes based on presence/absence of bacterial taxa might be explained by the higher *P*. *grassii* protozoa counts in male compared to female guts. Higher protozoa numbers may result in more niches for bacteria in male guts, which is supported by the observed correlation of bacteria richness and abundance with *P*. *grassi* and total protozoa numbers, respectively. This especially pertains to the protozoa associated bacterial symbionts, whose numbers correlated with the numbers of their putative protozoa host. Moreover, the explanations for higher *P*. *grassii* numbers in males also apply to the increased bacteria diversity and abundance, including male-biased nutrition contributions [35, 82, 86]. Although most colonies produce a female or male biased sex ratio in alates to reduce inbreeding [37], colony origin and sex-biased alate production are not likely causes of the sex bias in bacteria numbers, since this occurred in most samples and all populations.

In contrast to weight and protozoa numbers, neither bacterial alpha nor beta diversity had any effect on pair formation. This suggests that the bacteria community at the time of colony initiation is of less nutritional importance than the proteins and lipids provided by protozoa and the alates’ fat body. Nevertheless, our study showed that the core phyla and expected dominant taxa were present in dealates as “starter package” to inoculate the first brood, regardless of bacterial diversity. Follow-up studies are currently underway to investigate how bacterial communities in founder pairs change over the course of early colony development and if their dynamics is associated with observed spikes in protozoa numbers [110].

### Do dealates assess the quality of potential partners based on weight and microbiota?

The observed differences in weight and protozoa numbers between paired and unpaired delates raise the question of whether active mate choice is at play and how dealates could recognize these differences in potential partners. Alternatively, high weight and protozoa numbers could confer competitive advantage for getting access to partners which would not necessarily require partner assessment.

Weight can be easily assessed visually or by antennation during tandem-running and some studies have reported a positive correlation in size and/or weight between partners [41, 85]. However, our present study found no correlation in weights between partners in established nest chambers and weight could not serve as a proxy to assess the quality or quantity of gut microbiota in *C*. *formosanus* dealates, since no correlation was observed between weight and protozoa numbers or between weight and bacteria diversity. Similar to the results for weight, protozoa numbers and *P*. *grassii* percentage were also higher in paired dealates with no correlation among partners.

The lack of correlation of the respective weights and protozoa numbers between partners suggests that the observed prevalence of dealates with larger weight and protozoa numbers in pairs might not be caused by stringent mutual mate choice, i.e., weight–and symbiont-biased assessment of potential partners. Moreover, predators and environmental pressures likely select for rapid pair formation with limited opportunity to carefully assess and change partners [47, 50]. The fact that *C*. *formosanus* and *C*. *gestroi* males occasionally mate with females of the opposite species might also indicate that there is a lack of diligent partner assessment [43]. Instead, pair formation is likely driven by a competitive advantage in monopolizing partners gained by individuals with high weight and protozoa numbers. Dealates with heavy bodies and high numbers of protozoa can spend more energy to search for and obtain access to partners as well as outcompete rivals during tandem running since higher values represent better nutrition. For example, Mizumoto et al.’s [111] study showed that *C*. *gestroi* males increased their movement speed when searching for a partner in the presence of competition. Faster movement requires higher energy expenditure and, therefore, more fat and protein reserves.

## Supporting information

S1 File Metadata for all samples of *C*. *formosanus* dealates. (XLSX)

S2 File List of bacterial ASVs and their number of reads in all dealate gut samples. (XLSX)

S3 File This file contains S1 Fig. *Coptotermes formosanus* alate collection in the field and paired dealate collection from corrugated cardboard nest chambers. S2 Fig. Sequence-based rarefaction curves of the bacterial diversity of each sample measured by ASV richness, Faith’s PD and Shannon indices. S3 Fig. Sample- and coverage-based rarefaction curves across all dealate samples.
(DOCX)

S4 File This file contains S1 Table. Dealates’ weight, total protozoa number, *P*. *grassii* number and percentage of *P*. *grassii* separated by pairing status, sex, and population. S2 Table. Spearman’s rank correlations for dealate weights and protozoa counts. S3 Table. Nineteen bacterial phyla detected across all dealate samples. S4 Table. Correlation analysis (Spearman’s rank test) of protozoa abundance in *C*. *formosanus* dealates with rarefied number of reads of previously reported putative symbiotic bacteria of protozoa in termites. S5 Table. Impact of pairing status, sex, and population on alpha diversity of dealate gut bacterial communities. S6 Table. Correlation of dealates’ weight, total protozoa number, *P*. *grassii* number, and percentage of *P*. *grassii* protozoa to bacterial alpha diversity metrics. S7 Table. Effects of pairing status, sex and population on bacterial beta diversity of dealates. S8 Table. Single factor beta diversity analyses using PERMANOVA (999 permutations) and PERMDISP (1000 permutations).
(DOCX)
