# Supplementary material for: Weight and protozoa number but not bacteria diversity are associated with successful pair formation of dealates in the Formosan subterranean termite, Coptotermes formosanus
Source: PLoS One. 2023 Nov 13;18(11):e0293813. doi: 10.1371/journal.pone.0293813 (PMC10642788; doi:10.1371/journal.pone.0293813)

**Figure S1: *Coptotermes formosanus* alate collection in the field and paired dealate collection from corrugated cardboard nest chambers**. During swarm events light traps were set out at four different locations consisting of an 18.9 L bucket with a ring light placed on a white mat. Corrugated cardboard pieces were randomly placed inside and outside the bucket. Alates can be seen landing on the mat (pictures on left). Alates and cardboard pieces were put into large containers in the lab overnight. The next morning, paired dealates were collected from inside sealed cardboard chambers after tearing off the paper layer (pictures on right).


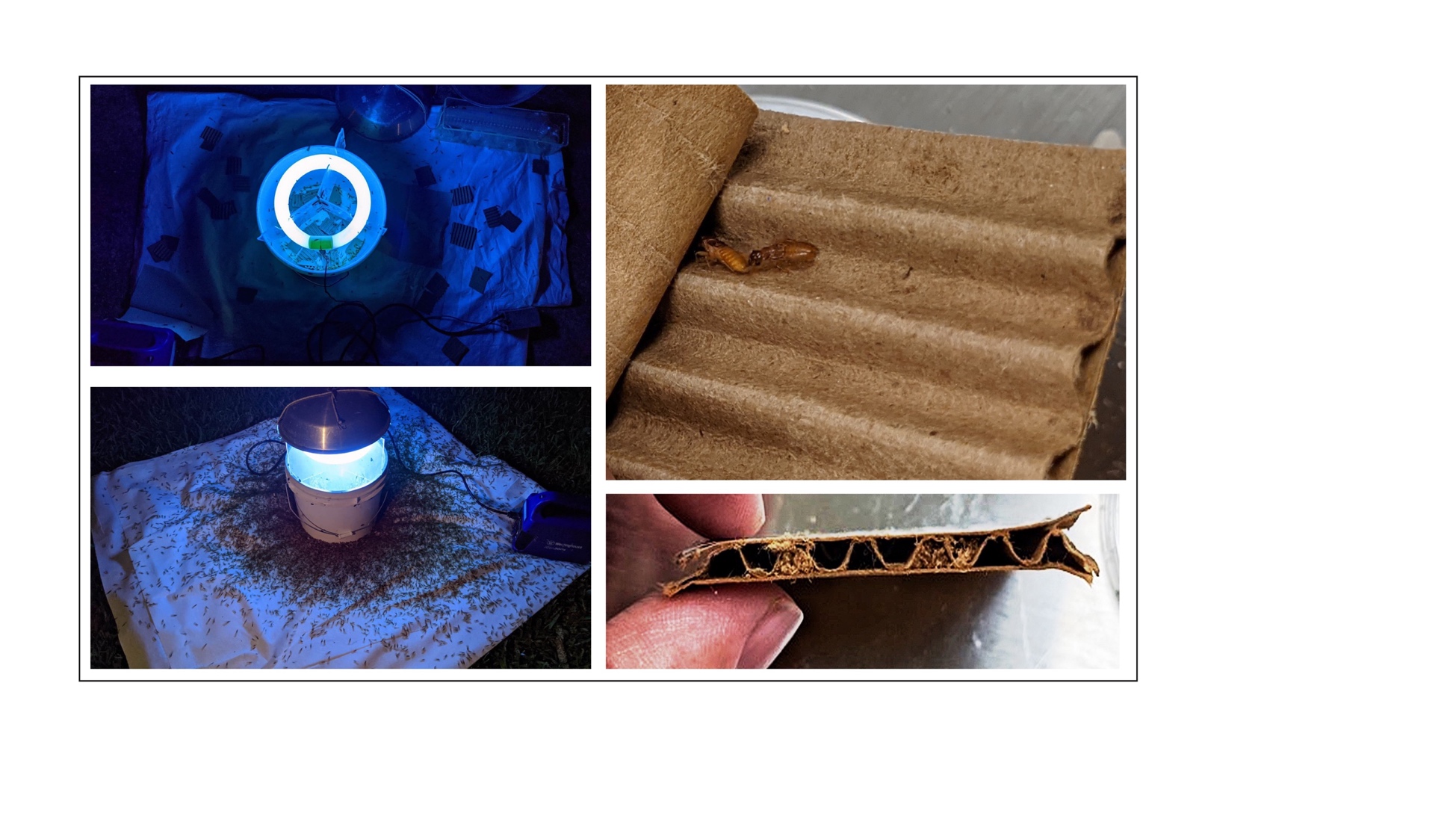


**Figure S2: Sequence-based rarefaction curves of the bacterial diversity of each sample measured by ASV richness, Faith’s PD and Shannon indices.** Over 3 million raw sequence reads were generated from 80 samples representing 1,489 ASVs. Rarefaction curves for ASV numbers, Faith’s PD and Shannon diversity plateaued for all samples at a sequencing depth of less than 892.


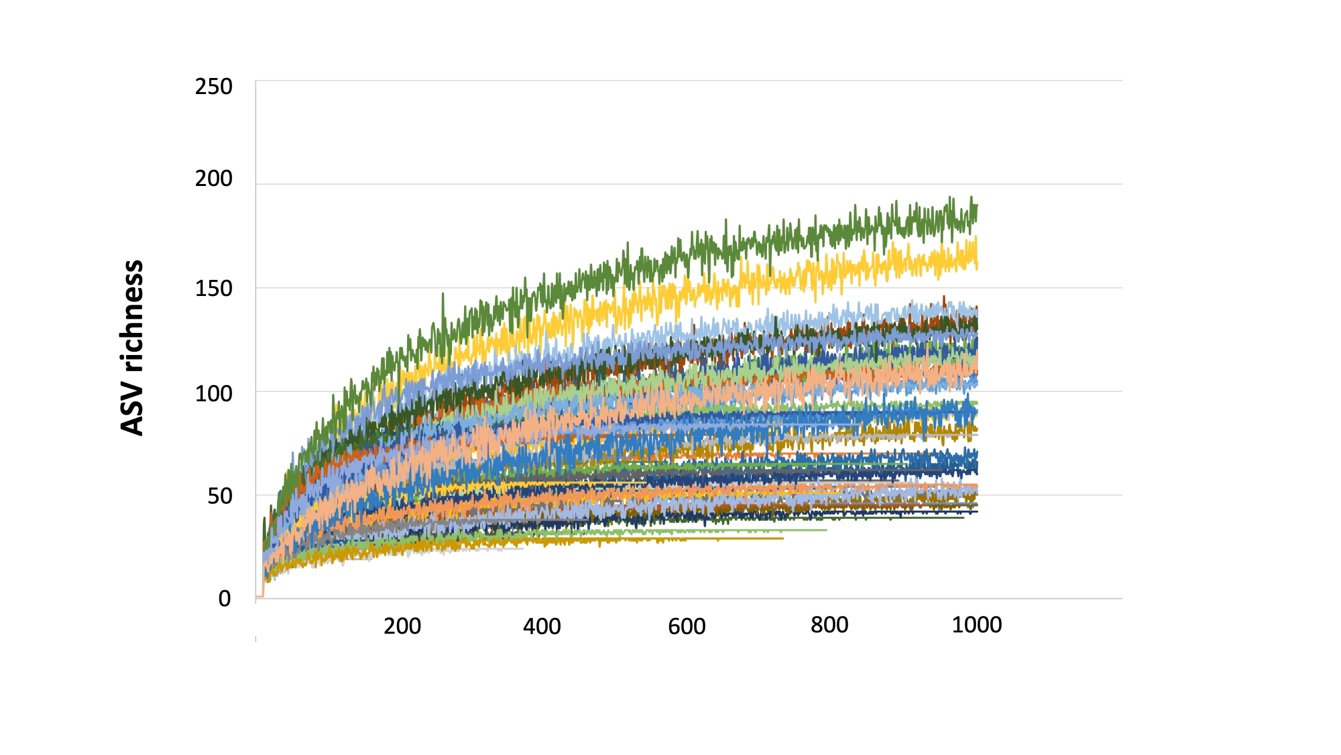


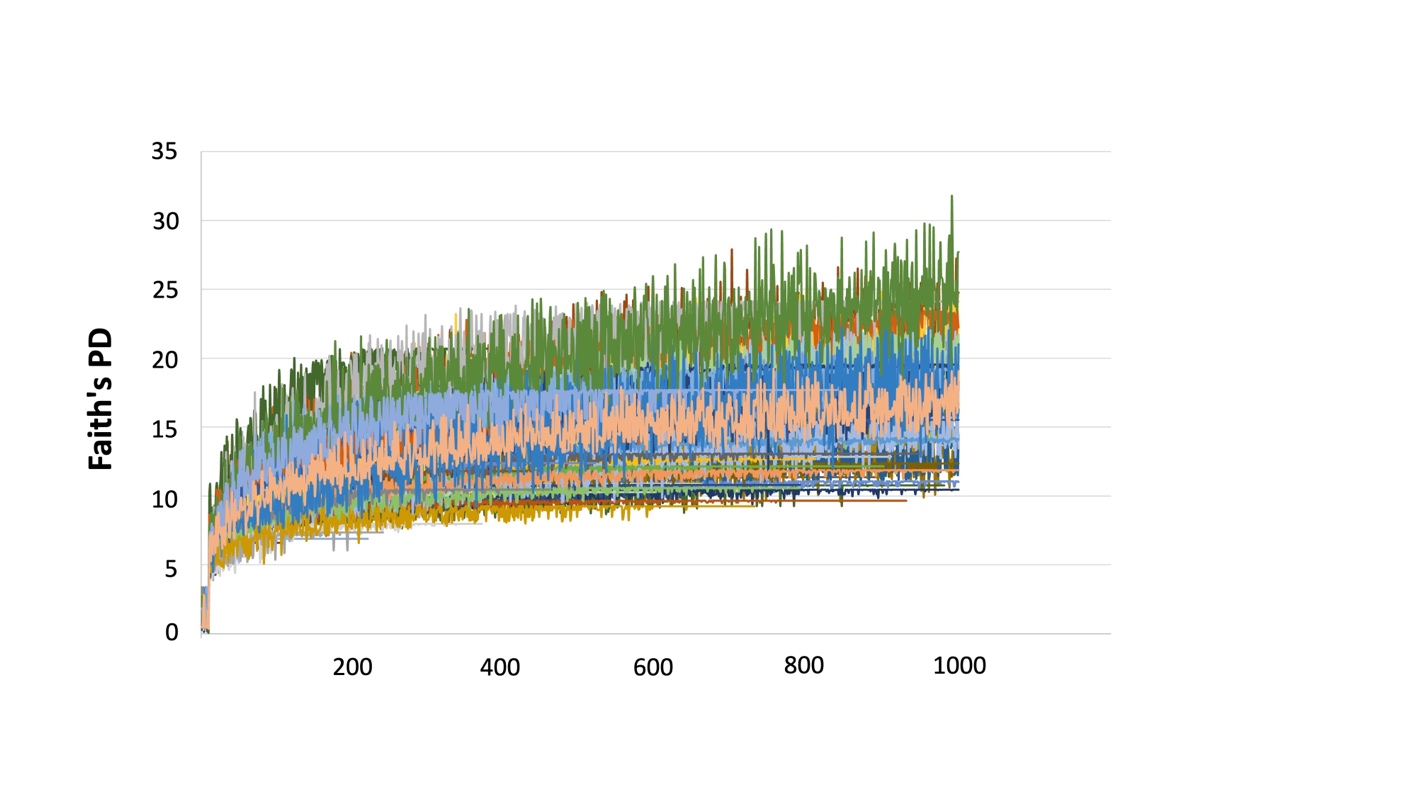


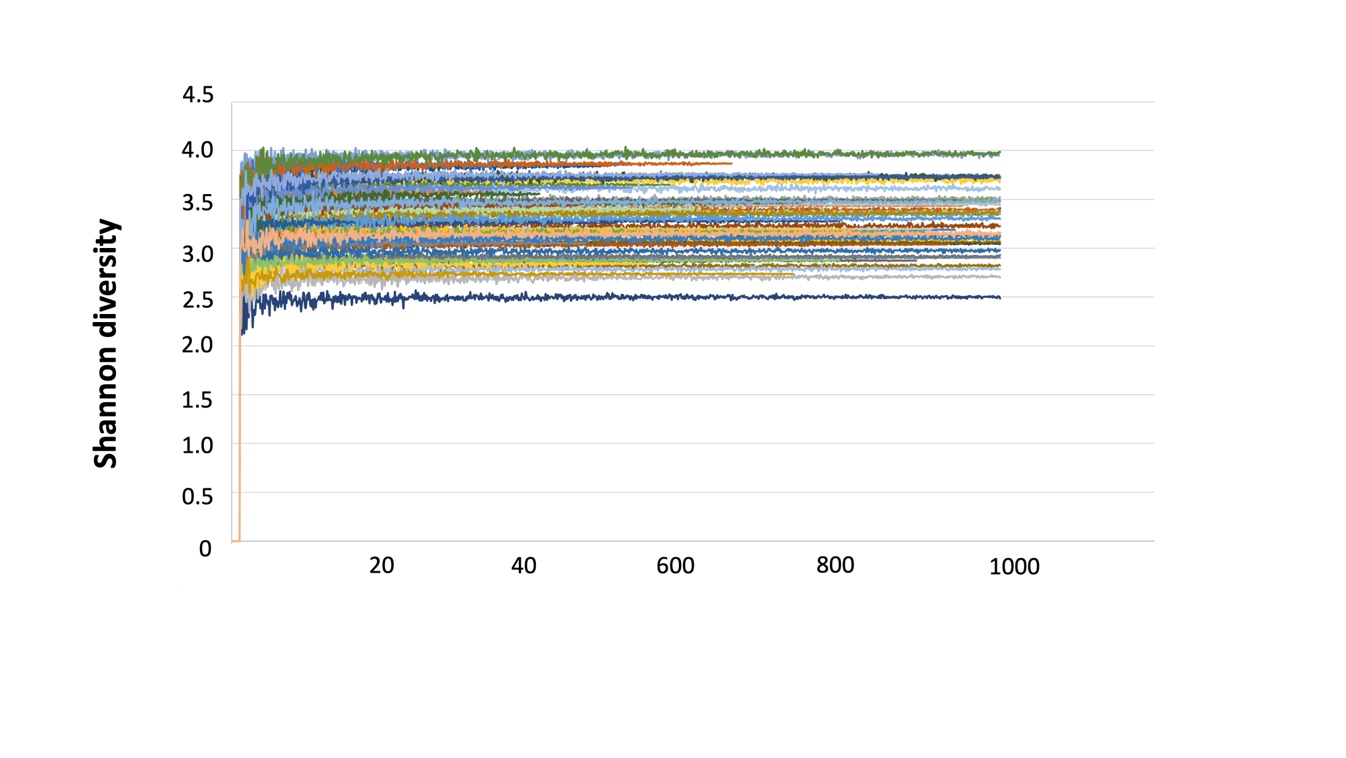


**Figure S3. Sample- and coverage-based rarefaction curves across all dealate samples.** Sample-based rarefaction curves with effective bacterial diversity for different metrics plotted against the number of samples. (B). Coverage-based rarefaction curves with effective diversity plotted against estimated sample coverage The solid lines in the figure represent the interpolated results up to the actual sample size (n=80), while the dashed lines represent the extrapolation to twice the sample size. The 95% confidence interval is shown by the shaded area around each curve.


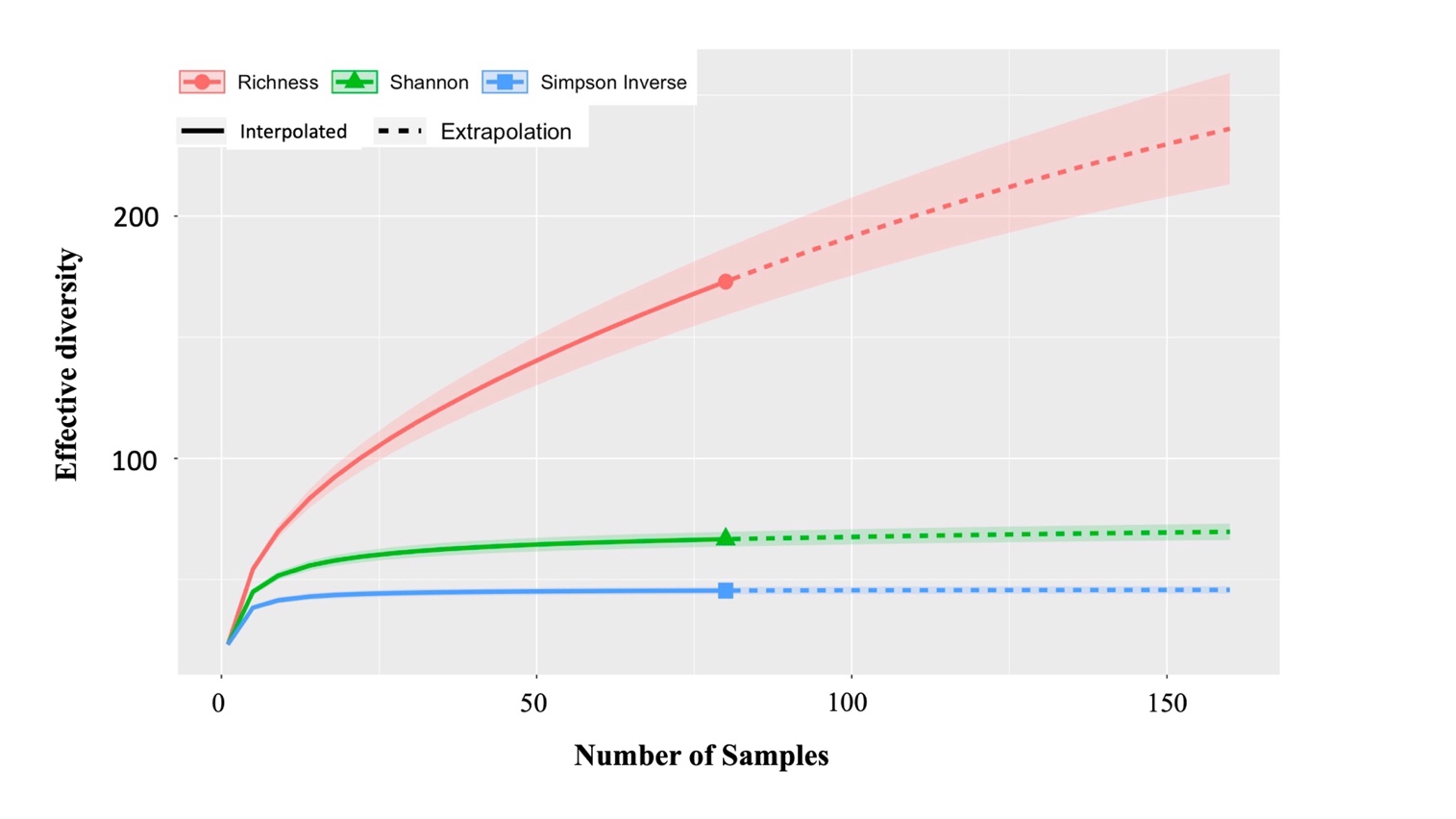


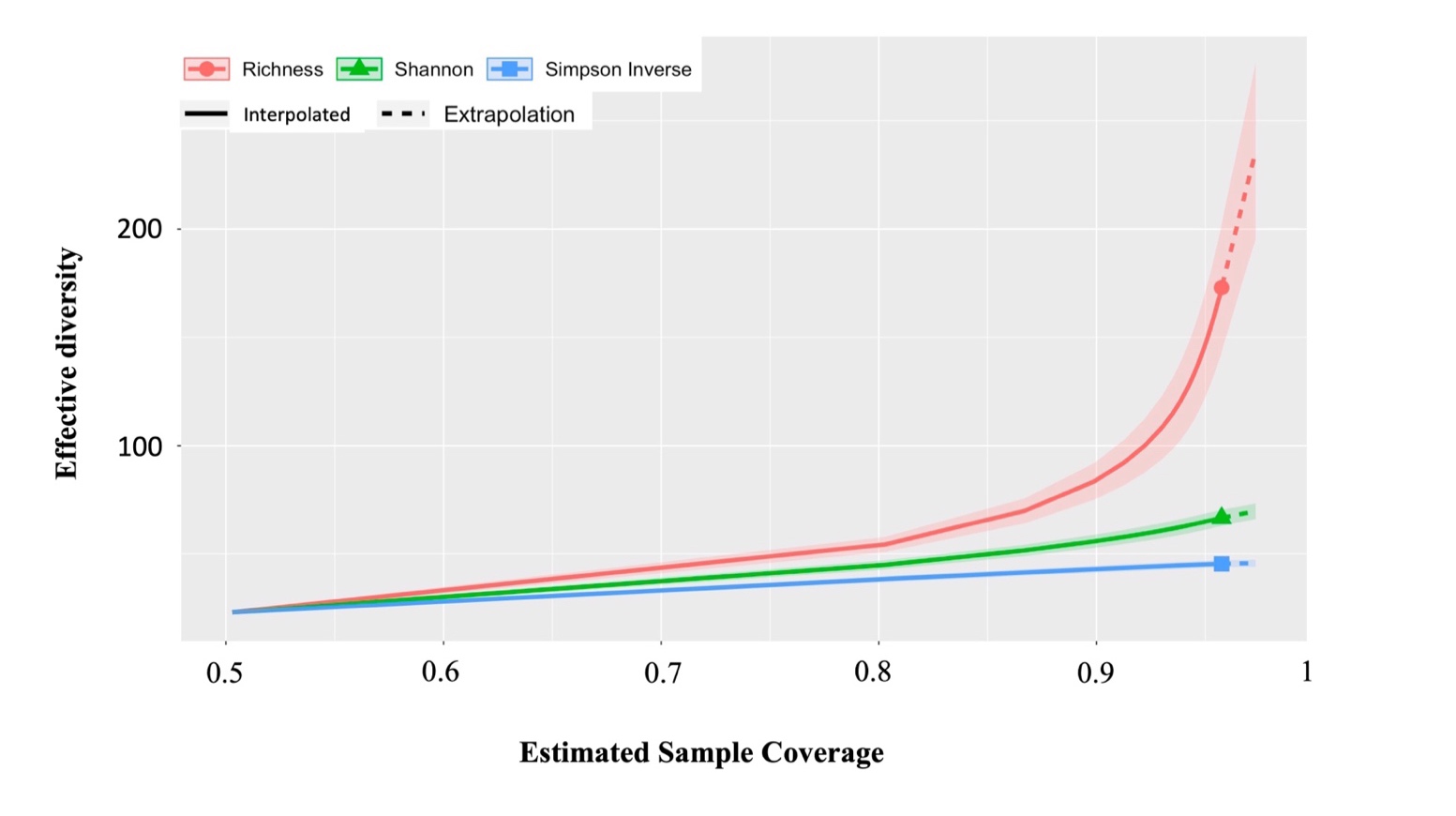

Supplement: S3 File — Coptotermes formosanus alate collection in the field and paired dealate collection from corrugated cardboard nest chambers. S2 Fig. Sequence-based rarefaction curves of the bacterial diversity of each sample measured by ASV richness, Faith’s PD and Shannon indices. S3 Fig. Sample- and coverage-based rarefaction curves across all dealate samples. (DOCX) [file pone.0293813.s003.docx]
