# Supplementary material for: Weight and protozoa number but not bacteria diversity are associated with successful pair formation of dealates in the Formosan subterranean termite, Coptotermes formosanus
Source: PLoS One. 2023 Nov 13;18(11):e0293813. doi: 10.1371/journal.pone.0293813 (PMC10642788; doi:10.1371/journal.pone.0293813)
Supplement: S4 File — Dealates’ weight, total protozoa number, P. grassii number and percentage of P. grassii separated by pairing status, sex, and population. S2 Table. Spearman’s rank correlations for dealate weights and protozoa counts. S3 Table. Nineteen bacterial phyla detected across all dealate samples. S4 Table. Correlation analysis (Spearman’s rank test) of protozoa abundance in C. formosanus dealates with rarefied number of reads of previously reported putative symbiotic bacteria of protozoa in termites. S5 Table. Impact of pairing status, sex, and population on alpha diversity of dealate gut bacterial communities. S6 Table. Correlation of dealates’ weight, total protozoa number, P. grassii number, and percentage of P. grassii protozoa to bacterial alpha diversity metrics. S7 Table. Effects of pairing status, sex and population on bacterial beta diversity of dealates. S8 Table. Single factor beta diversity analyses using PERMANOVA (999 permutations) and PERMDISP (1000 permutations). (DOCX) [file pone.0293813.s004.docx]

**Table S1: Dealates’ weight, total protozoa number, *P. grassii* number and percentage of *P. grassii* separated by pairing status, sex, and population.** Means and standard deviations (SD) of each group in each factor were calculated. Groups were first tested for significant differences by univariate tests, i.e., two-tailed, unpaired t-tests with Welch’s correction for pairing status and sex and with ANOVA for population. Significances and interactions of the factors were further tested with multivariate analysis of variance (MANOVA). A factor was only considered to be significant when both MANOVA and univariate tests showed significance (P < 0.05). Interactions were not calculated for single factor analyses and were marked as not applicable (NA).

Pairing status had a significant effect on weight, total protozoa count, and the percentage of *P. grassii*. Sex had a significant effect on weight and *P. grassii* counts, while population had a significant effect on total protozoa number. The only significant interaction was observed between pairing status and population for the percentage of *P. grassii*.

| **Factors** | **Weight** ± **SD (mg)** | **Univariate**  **P-value** | **MANOVA P-value** | **Significant** | **Total protozoa number** ± **SD** | **Univariate**  **P-value** | **MANOVA P-value** | **Significant** |
| --- | --- | --- | --- | --- | --- | --- | --- | --- |
|  |  |  |  |  |  |  |  |  |
| **Pairing Status** |  |  |  |  |  |  |  |  |
| Paired | 9.82 ± 0.60 | **< 0.0001** | **8.21E-11** | **Yes** | 974.4 ± 384.87 | **< 0.0001** | **3.33E-06** | **Yes** |
| Unpaired | 8.96 ± 0.79 |  |  |  | 646.5 ± 255.63 |  |  |  |
| **Sex** |  |  |  |  |  |  |  |  |
| Female | 9.85 ± 0.71 | **< 0.0001** | **1.33E-11** | **Yes** | 749.9 ± 355.01 | 0.1381 | 0.0646 | No |
| Male | 8.93 ± 0.65 |  |  |  | 870.9 ± 367.52 |  |  |  |
| **Population** |  |  |  |  |  |  |  |  |
| BB | 9.17 ± 0.77 | 0.3036 | **0.0334** | No | 1063.33 ± 412.70 | **0.0089** | **0.0002** | **Yes** |
| BG | 9.35 ± 0.82 |  |  |  | 692.08 ± 299.32 |  |  |  |
| NO | 9.40 ± 0.91 |  |  |  | 676.25 ± 307.04 |  |  |  |
| SG | 9.64 ± 0.77 |  |  |  | 810.00 ± 310.10 |  |  |  |
| **Pairing Status: Sex** | NA | NA | 0.8230 | No | NA | NA | 0.2969 | No |
| **Pairing Status: Population** | NA | NA | 0.0507 | No | NA | NA | 0.5043 | No |
| **Sex: Population** | NA | NA | 0.6576 | No | NA | NA | 0.6472 | No |
| **Pairing Status: Sex: Population** | NA | NA | 0.3478 | No | NA | NA | 0.2132 | No |
| **Factors** | ***P. grassii* number** ± **SD** | **Univariate**  **P-value** | **MANOVA P-value** | **Significant** | ***P. grassii* percentage** ± **SD (%)** | **Univariate**  **P-value** | **MANOVA P-value** | **Significant** |
|  |  |  |  |  |  |  |  |  |
| **Pairing Status** |  |  |  |  |  |  |  |  |
| Paired | 125.0 ± 75.68 | 0.6889 | 0.6828 | **No** | 11.97 ± 5.84 | **0.0004** | **0.0001** | **Yes** |
| Unpaired | 133.1 ± 79.34 |  |  |  | 19.75 ± 9.44 |  |  |  |
| **Sex** |  |  |  |  |  |  |  |  |
| Female | 105.6 ± 64.12 | **0.017** | **0.0205** | **Yes** | 13.82 ± 8.04 | 0.0693 | **0.0318** | No |
| Male | 152.5 ± 82.50 |  |  |  | 17.90 ± 9.01 |  |  |  |
| **Population** |  |  |  |  |  |  |  |  |
| BB | 137.92 ± 86.03 | 0.3339 | 0.3189 | No | 12.59 ± 5.92 | 0.0685 | **0.0256** | No |
| BG | NA |  |  |  | NA |  |  |  |
| NO | 107.92 ± 81.85 |  |  |  | 16.05 ± 9.40 |  |  |  |
| SG | 141.25 ± 59.94 |  |  |  | 18.94 ± 9.54 |  |  |  |
| **Pairing Status: Sex** | NA | NA | 0.8230 | No | NA | NA | 0.607 | No |
| **Pairing Status: Population** | NA | NA | 0.0507 | No | NA | NA | **0.0247** | **Yes** |
| **Sex: Population** | NA | NA | 0.6576 | No | NA | NA | 0.824 | No |
| **Pairing Status: Sex: Population** | NA | NA | 0.3478 | No | NA | NA | 0.7862 | No |

**Table S2. Spearman’s rank correlations for dealate weights and protozoa counts.**

A: Lack of correlation between partners of each pair of dealates for weight and all protozoa counts.

B: Correlations among weight and the three protozoa counts for all dealates.

Significant P-values (<0.05) are indicated in bold font. Positive and negative correlations are indicated by the Spearman’s rank coefficient Rho, which ranges from -1 to 1.

| 1. **Between partners** | **Weight** | | **Total protozoa number** | | ***P. grassii* number** | | **Percentage of *P. grassii*** | |
| --- | --- | --- | --- | --- | --- | --- | --- | --- |
|  | P-value | Rho | P-value | Rho | P-value | Rho | P-value | Rho |
|  | 0.9746 | -0.0076 | 0.1030 | 0.3752 | 0.3001 | 0.2867 | 0.1824 | -0.3643 |
|  |  |  |  |  |  |  |  |  |
|  |  |  |  |  |  |  |  |  |
| 1. **Among weight and protozoa counts** | **Weight** | | **Total protozoa number** | | ***P. grassii* number** | | **Percentage of *P. grassii*** | |
|  | P-value | Rho | P-value | Rho | P-value | Rho | P-value | Rho |
| **Weight** | - | - | 0.1140 | 0.1781 | 0.6092 | -0.0673 | 0.0620 | -0.2424 |
| **Total protozoa number** |  |  | - | - | **0.0001** | 0.4804 | 0.3238 | -0.1296 |
| ***P. grassii* number** |  |  |  |  | - | - | **1.15E-12** | 0.7647 |
|  |  |  |  |  |  |  |  |  |

**Table S3. Nineteen bacterial phyla detected across all dealate samples.** Bacterial phyla were ranked from highest to lowest according to numbers of reads and relative abundance and presented along with their number of ASVs and the number of dealate samples they were detected in.

| **Phylum** | **Number of reads** | **Percentage of total assigned bacterial reads (%)** | **Number of ASVs in phylum** | **Number of dealates** |
| --- | --- | --- | --- | --- |
| Bacteroidota | 150,283 | 50.28 | 80 | 80 |
| Proteobacteria | 63,379 | 21.21 | 113 | 80 |
| Firmicutes | 39,023 | 13.06 | 101 | 80 |
| Spirochaetota | 34,146 | 11.43 | 218 | 80 |
| Desulfobacterota | 4,216 | 1.41 | 19 | 71 |
| Actinobacteriota | 3,192 | 1.07 | 41 | 59 |
| Campilobacterota | 2,014 | 0.68 | 3 | 27 |
| Synergistota | 1,736 | 0.58 | 17 | 33 |
| Planctomycetota | 373 | 0.12 | 12 | 36 |
| Verrucomicrobiota | 251 | 0.08 | 3 | 25 |
| Elusimicrobiota | 99 | 0.03 | 3 | 8 |
| Cyanobacteria | 93 | 0.03 | 6 | 9 |
| Deferribacterota | 26 | <0.01 | 1 | 2 |
| Margulisbacteria | 17 | <0.01 | 1 | 3 |
| Fibrobacterota | 7 | <0.01 | 2 | 2 |
| Acidobacteriota | 3 | <0.01 | 1 | 1 |
| Armatimonadota | 2 | <0.01 | 1 | 1 |
| Bdellovibrionota | 2 | <0.01 | 1 | 1 |
| Deinococcota | 2 | <0.01 | 1 | 1 |
|  |  |  |  |  |
| Total | 298,864 | 100 | 624 | 80 |

**Table S4: Correlation analysis (Spearman’s rank test) of protozoa abundance in *C. formosanus* dealates with rarefied number of reads of previously reported putative symbiotic bacteria of protozoa in termites.** Significant values are highlighted in bold.

|  | ***Ca*. Azobacteroides** | |
| --- | --- | --- |
|  | P-value | Rho |
| **Total Protozoa number** | **0.0135** | 0.2752 |
| ***P. grassii* number** | **0.0004** | 0.4391 |
| ***P. grassii* %** | 0.0717 | 0.2342 |
|  | ***Ca*. Armantifilum** | |
|  | P-value | Rho |
| **Total Protozoa number** | **0.0367** | 0.2340 |
| ***P. grassii* number** | 0.2075 | 0.1651 |
| ***P. grassii* %** | 0.9452 | -0.0090 |
|  | ***Ca*. Vestibaculum** | |
|  | P-value | Rho |
| **Total Protozoa number** | 0.4348 | -0.0885 |
| ***P. grassii* number** | 0.226 | 0.1587 |
| ***P. grassii* %** | 0.0729 | 0.234 |

**Table S5. Impact of pairing status, sex, and population on alpha diversity of dealate gut bacterial communities.** No significant differences were detected between paired and unpaired alates. Significant differences (in bold font) were observed in ASV numbers between males and females and in phylogenetic distances (Faith’s PD) among the bacterial communities of different populations (Kruskal-Wallis test).

| **Alpha diversity metrics** | **Factors** | **P-value** |
| --- | --- | --- |
| ASV numbers | Pairing Status | 0.9463 |
|  | Sex | **0.0484** |
|  | Population | 0.1555 |
| Shannon diversity | Pairing Status | 0.4587 |
|  | Sex | 0.6168 |
|  | Population | 0.0931 |
| Pielou’s evenness | Pairing Status | 0.3506 |
|  | Sex | 0.9616 |
|  | Population | 0.1158 |
| Faith’s PD | Pairing Status | 0.2404 |
|  | Sex | 0.0606 |
|  | Population | **0.0188** |

**Table S6. Correlation of dealates’ weight, total protozoa number, *P. grassii* number, and percentage of *P. grassii* protozoa to bacterial alpha diversity metrics.** Significant correlations (Spearman-rank test, P < 0.05) were marked in bold.

| **Alpha diversity metrics** | **Factors** | **Spearman-rank test** | | **Sample size** |
| --- | --- | --- | --- | --- |
|  |  | **P-value** | **Rho** |  |
| ASV richness  (number of ASVs) | Weight | 0.5956 | -0.0602 | 80 |
|  | Total Protozoa number | 0.1349 | -0.1686 | 80 |
|  | Number of *P. grassii* | **0.0418** | **0.2637** | 60 |
|  | Percentage of *P. grassii* | 0.1201 | 0.2028 | 60 |
| ASV abundance (number of reads) | Weight | 0.4288 | -0.897 | 80 |
|  | Total Protozoa number | **0.0487** | **0.2211** | 80 |
|  | Number of *P. grassii* | 0.3914 | 0.1127 | 60 |
|  | Percentage of *P. grassii* | 0.7100 | -0.049 | 60 |
| Shannon diversity | Weight | 0.7385 | 0.0379 | 80 |
|  | Total Protozoa number | 0.0947 | -0.1881 | 80 |
|  | Number of *P. grassii* | 0.2168 | 0.1618 | 60 |
|  | Percentage of *P. grassii* | 0.0625 | 0.2420 | 60 |
| Pielou’s evenness | Weight | 0.5970 | 0.0600 | 80 |
|  | Total Protozoa number | 0.0876 | -0.1922 | 80 |
|  | Number of *P. grassii* | 0.2560 | 0.1490 | 60 |
|  | Percentage of *P. grassii* | 0.0604 | 0.2439 | 60 |
| Faith’s PD | Weight | 0.6649 | -0.0492 | 80 |
|  | Total Protozoa number | 0.2985 | -0.1177 | 80 |
|  | Number of *P. grassii* | 0.9252 | -0.0124 | 60 |
|  | Percentage of *P. grassii* | 0.6871 | 0.0531 | 60 |

**Table S7. Effects of pairing status, sex and population on bacterial beta diversity of dealates.** ADONIS (multifactorial PERMANOVA) tests were performed using four different beta diversity indices. Significant values are highlighted in bold.

|  | **Weighted Unifrac** | | **Bray Curtis** | |
| --- | --- | --- | --- | --- |
|  | **R2** | **Pr(>F)** | **R2** | **Pr(>F)** |
| **Pairing Status** | 0.0024 | 0.8180 | 0.0079 | 0.5460 |
| **Sex** | 0.0069 | 0.5190 | 0.0111 | 0.2940 |
| **Population** | 0.1270 | **0.0060** | 0.1444 | **0.0010** |
| **Population: Sex** | 0.0519 | 0.1820 | 0.0568 | 0.0960 |
| **Population: Pairing Status** | 0.0126 | 0.8850 | 0.0176 | 0.8790 |
| **Sex: Pairing Status** | 0.0057 | 0.5940 | 0.0122 | 0.3020 |
| **Population: Sex: Pairing Status** | 0.0694 | 0.0710 | 0.0341 | 0.4410 |
| **Residuals** | 0.7242 | NA | 0.7158 | NA |
| **Total** | 1.0000 | NA | 1.0000 | NA |
|  |  |  |  |  |
|  | **Unweighted Unifrac** | | **Jaccard** | |
|  | **R2** | **Pr(>F)** | **R2** | **Pr(>F)** |
| **Pairing Status** | 0.0120 | 0.3630 | 0.0116 | 0.4640 |
| **Sex** | 0.0197 | 0.0550 | 0.0181 | **0.0080** |
| **Population** | 0.0760 | **0.0010** | 0.0843 | **0.0010** |
| **Population: Sex** | 0.0424 | 0.1470 | 0.0405 | 0.0860 |
| **Population: Pairing Status** | 0.0363 | 0.4090 | 0.0387 | 0.1560 |
| **Sex: Pairing Status** | 0.0263 | **0.0120** | 0.0174 | **0.0130** |
| **Population: Sex: Pairing Status** | 0.0409 | 0.2100 | 0.0363 | 0.3620 |
| **Residuals** | 0.7464 | NA | 0.7531 | NA |
| **Total** | 1.0000 | NA | 1.0000 | NA |

**Table S8. Single factor beta diversity analyses using PERMANOVA (999 permutations) and PERMDISP (1000 permutations)**. Distance matrices were calculated with Weighted UniFrac, Bray Curtis, Unweighted UniFrac and Jaccard metrics. P-values < 0.05 after Benjamini-Hochberg correction were considered significant and were bolded.

|  | **Weighted Unifrac** | | **Bray Curtis** | |
| --- | --- | --- | --- | --- |
|  | PERMANOVA | PERMDISP | PERMANOVA | PERMDISP |
| **Pairing Status** | 0.879 | 0.4685 | 0.665 | 0.8262 |
| **Sex** | 0.58 | 0.6699 | 0.369 | 0.6483 |
| **Population** | **0.004** | **0.006** | **0.005** | **0.005** |
|  |  |  |  |  |
|  | **Unweighted Unifrac** | | **Jaccard** | |
|  | PERMANOVA | PERMDISP | PERMANOVA | PERMDISP |
| **Pairing Status** | 0.482 | 0.5674 | 0.655 | 0.6593 |
| **Sex** | 0.061 | 0.6535 | **0.016** | 0.9251 |
| **Population** | 0.0859 | 0.0859 | **0.009** | **0.009** |
